# The giant eyes of giant squid are indeed unexpectedly large, but not if used for spotting sperm whales

**DOI:** 10.1186/1471-2148-13-187

**Published:** 2013-09-08

**Authors:** Dan-E Nilsson, Eric J Warrant, Sönke Johnsen, Roger T Hanlon, Nadav Shashar

**Affiliations:** 1 Department of Biology, Lund University, Lund 22362, Sweden; 2 Biology Department, Duke University, Durham, NC 27708, USA; 3 Marine Biological Laboratory, Woods Hole, MA 02543, USA; 4 Department of Life Sciences, Eilat Campus, Ben Gurion University of the Negev, Eilat 88000, Israel

**Keywords:** Vision, Eyes, Giant squid, Sperm whale, Bioluminescence, Mesopelagic

## Abstract

**Background:**

We recently reported (Curr Biol 22:683–688, 2012) that the eyes of giant and colossal squid can grow to three times the diameter of the eyes of any other animal, including large fishes and whales. As an explanation to this extreme absolute eye size, we developed a theory for visual performance in aquatic habitats, leading to the conclusion that the huge eyes of giant and colossal squid are uniquely suited for detection of sperm whales, which are important squid-predators in the depths where these squid live. A paper in this journal by Schmitz et al. (BMC Evol Biol 13:45, 2013) refutes our conclusions on the basis of two claims: (1) using allometric data they argue that the eyes of giant and colossal squid are not unexpectedly large for the size of the squid, and (2) a revision of the values used for modelling indicates that large eyes are not better for detection of approaching sperm whales than they are for any other task.

**Results and conclusions:**

We agree with Schmitz et al. that their revised values for intensity and abundance of planktonic bioluminescence may be more realistic, or at least more appropriately conservative, but argue that their conclusions are incorrect because they have not considered some of the main arguments put forward in our paper. We also present new modelling to demonstrate that our conclusions remain robust, even with the revised input values suggested by Schmitz et al.

## Information from allometry versus functional theory

Schmitz et al. [1] explicitly assume that eyes are “expected” to scale allometrically with body size. But there is no known biological reason why eye size and body size should scale according to a power law with a constant exponent over several orders of magnitude. The purpose of allometry is to describe scaling relationships, not to explain them. In contrast, our paper [2] is based on functional arguments, and reveals a law of diminishing returns, which renders it less rewarding to continue increasing the eye size the larger an eye becomes. This law of diminishing returns is caused by absorption and scattering of light in water, and is highly relevant for eye sizes that range from a few cm to the huge eyes of giant squid.

As an example, at 300 m depth, dark objects can be seen at a 6.5% longer range if the diameter of a 1 mm eye is increased by 10%, but if the eye is already 10 mm in diameter, a 10% further increase in diameter will only generate a 4% longer visual range, and for a 100 mm eye, a 10% size increase improves the visual range only by a modest 2.5%. The eyes of giant and colossal squid grow to almost 3 times this diameter, even though this provides a relatively small improvement in visual range. These theoretical findings strongly suggest that eyes of very large animals should be relatively smaller, given that the energetic cost of eyes scales linearly with body mass.

Schmitz et al. [1] found that the eye size of adult giant squid is on or above the linear regression line on a log-log plot representing the scaling relationship for 87 smaller squid species. Because the exponent of the power law is below 1, allometry does indeed reveal a general tendency for larger squid to have relatively smaller eyes, as predicted by our law of diminishing returns. A striking feature of their results is the huge variation, which, for any given body size, allows a more than 6-fold difference in eye diameter within the standard deviation. This large variation undoubtedly reflects major differences in the visual ecology of different squid species, and speaks against the conclusion of Schmitz et al. [1] that eye size is developmentally constrained in squid.

## The unique visual task for giant squid

**Figure 1:**
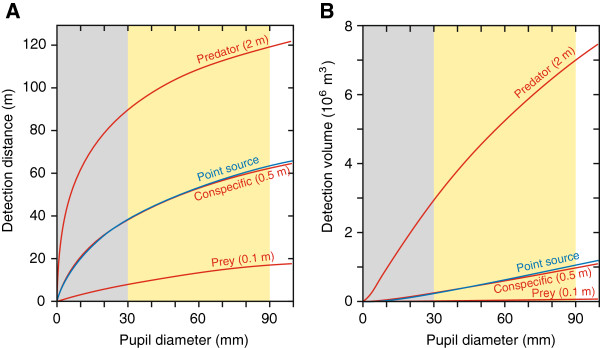
**Modelling visual performance in relation to eye size.** Calculations are made for a depth of 800 m in clear oceanic water where the lack of daylight makes visual performance independent of viewing direction. Performance is plotted against pupil diameter (approximately a third of the eye diameter). Calculations are made for detection of bioluminescent point sources (blue) and for extended luminous targets (red) of different sizes corresponding to prey animals, conspecifics and predators (sperm whales) [2]. For bioluminescent flash intensity (*E*) and nearest neighbour distance (*x*) we use the alternative values of Schmitz et al. [1]: *E* =10^10^ quanta/s, *x* = 55 cm. All calculations are made using the theory of Nilsson et al. [2], but for extended targets, the calculations were modified such that the number of point sources within the target pixel is calculated in 3 dimensions for the volume of water displaced by the moving target (a cylinder with target diameter and a length of 2.5 times that diameter). The density of luminous plankton (per unit volume) is 0.7/x^3^[3]. In the original calculations [1,2], a 2-dimensional case was considered, where the density of luminous plankton in the target pixel was calculated as 1/4x^2^[4]. **A**. Performance plotted as maximum detection distance, revealing that objects the size of sperm whales can be detected at nearly 120 m distance, which is almost twice the maximum detection distance for any other visible objects. **B**. Performance plotted as the volume of water in which objects can be detected. Detection of sperm whales massively outperforms all other visual functions, and the slope of the predator curve indicates that it offers a superior return for increasing eye size throughout the unique size range of giant and colossal squid (yellow shading). Blue shading indicates the size range of eyes in other animals.

In the light of the law of diminishing returns, we searched for possible reasons why the eyes of giant and colossal squid may grow to three times the absolute diameter of any other extant animal species. Such a reason was found by comparing different visual tasks. For recognition or pursuit of prey or mates, the range of vision is a good measure of performance, but for the likelihood of detecting rare but important objects, it is instead the volume of water within visible range that matters. Because the water volume has a cubic relation to visual range, it means that for such visual tasks, the law of diminishing returns is largely cancelled for all existing eye sizes, including those of giant squid (compare Figures 1A and B), which offers a potential explanation for extremely large eyes. Schmitz et al. [1] disregard this argument.

Our modelling of monitored water volume revealed that detection of very large extended objects provides much better performance returns than detection of smaller objects or point sources. Because giant and colossal squid live at depths where there is practically no daylight, the only large visible objects would be other animals triggering planktonic bioluminescence as they move through the water. The phenomenon is well known for revealing moving objects in the sea [5]. Because the performance return for increasing the eye size beyond that of the largest fish eyes (9–10 cm) and up to that of giant and colossal squid (27 cm) is much better for large than for small objects, we concluded that spotting the diffuse planktonic glow from their main predator, the sperm whale, offers a plausible and unique advantage that may have generated selection favouring huge eyes in giant and colossal squid.

## Revised values for modelling

Schmitz et al. [1] do not refute our theoretical reasoning for the unique advantage of giant eyes, but they argue that the values we used for modelling of triggered plankton bioluminescence were overestimates. With their alternative estimates of bioluminescent flash intensity and distance between luminous plankton, Schmitz et al. [1] found that our equations [2] no longer revealed any unique advantage for large eyes in detection of very large objects. But they did not take account of the fact that our equations were formulated such that they make a significant underestimate of the number of planktonic organisms that are triggered by moving targets.

The equations consider only a single layer of planktonic organisms within a circle inscribed within the target boundaries. In reality, the planktonic organisms that will be triggered to emit light are not restricted to a single layer, but involve the whole volume of water displaced by the moving target. For the prey of an approaching sperm whale, we can assume that at least the water displaced by the front 5 m of the whale’s linear length (to its widest point) will be in view. A conservative estimate of the displaced water volume seen by the prey would be a cylinder with a diameter of 2 m and a length of 5 m. If we use the revised value from Schmitz et al. [1] of 0.55 m for the nearest neighbour distance between luminous plankton, the displaced water volume in view will contain an average of 66 luminous plankton (for the relation between density and nearest neighbour distance in 3D see [3], and for the 2D case, see [4]). In the calculations of Schmitz et al. [1] the equivalent number is only 2.6 luminous plankton triggered by a 2 m target.

If we take the displaced water volume into account in the calculations of visual performance of very large eyes (Figure 1), even the revised lower values of plankton abundance and bioluminescent flash intensity suggested by Schmitz et al. [1] reverses their conclusion and thus supports our original findings [2]. Consequently, the assertions of Schmitz et al. [1] are insupportable and our suggestion that the huge eyes of giant squid are uniquely suited for detecting sperm whales remains intact.

## Competing interests

The authors have no competing interests to declare.

## References

[B1] Schmitz L Motani R Oufiero CE Martin CH McGee MD Gamarra AR Lee JJ Wainwright PC Allometry indicates that giant eyes of giant squid are not exceptional BMC Evol Biol 2013 13 45 10.1186/1471-2148-13-45 23418818 PMC3661360

[B2] Nilsson D-E Warrant EJ Johnsen S Hanlon R Shashar N A Unique Advantage for Giant Eyes in Giant Squid Curr Biol 2012 22 683 688 10.1016/j.cub.2012.02.031 22425154

[B3] Hertz P Über den gegenseitigen durchschnittlichen Abstand von Punkten, die mit bekannter mittlerer Dichte im Raume angeordnet sind Math Ann 1909 67 3 387 398 10.1007/BF01450410

[B4] Clark PJ Evans FC Distance to nearest neighbor as a measure of spatial relationships in populations Ecology 1954 35 445 453 10.2307/1931034

[B5] Herring PJ Dolphins glow with the flow Nature 1998 393 731 733 10.1038/31582

